# Dr. W. H. Dwinelle

**Published:** 1892-10

**Authors:** 


ARTICLE IV.

### DR. W. H. DWINELLE.

There is not an dentist in America who will not sigh with regret when he learns that the active days of usefulness of Dr. Dwinelle, of New York, are over. He has retired to his early home in Cazenovia, there to spend the last years of a long and honorable life in the quite retirement of a country home. What adds another pang to the general sorrow, is the knowledge that he is but inadequately supplied with money for his old age. He has always given as freely as he has received, and spent time and money lavishly in the service of the profession which he has so honored, and to which he has been so devoted. A few years ago, an unfortunate speculation swept away that which he had accumulated, and Dr. Dwinelle was left without sufficient provision for his old age.

We are not violating any of the rules of decorum in making these facts known, for it is through no misdeeds of his own that Dr. Dwinelle has met these misfortunes. Had he been of a selfish, grasping, avaricious nature, he might have been a rich man to-day, for the opportunities have been his to accumulate, had he not loved his profession and his professional brethren better than he loved himself. There are those occupying prominent positions to-day, who use those places for their own selfish ends. Dr. Dwinelle never did this, and his present poverty is a thousand times more honorable to him than would be the riches which have been attained by men who sacrified others to their own advancement.

There is not a dentist in America who is not in debt to Dr. Dwinelle. There is not one who is not reaping the benefits of his public profession or labors—who has not directly profited by that which was the result of Dr. Dwinelle's earnest study and self-sacrificing devotion to his profession. If he could receive but a tithe of this, his old age would be surrounded by everything that he could wish. Will not the dentists of New York State, especially, remember him now when his days of usefulness are numbered? At the very least, they can drop him a letter of sympathy and appreciation, to warm the heart which was never cold to any brother dentist's appeal. If they will enclose something a little more substantial, it will not be taken amiss. It is not charity. Let it be a token of goodwill and affectionate remembrance to one whose career has been honorable to himself and serviceable to his brother practitioners. How many will remember Dr. Dwinelle when the glad Thanksgiving Day shall come, and testify their gratitude and sympathy to one whose sympathies never yet slumbered when dentists or dentistry were concerned? He may be addressed at Cazenovia, N. Y., or if any one desires to unite with his brother dentists in doing something to smooth the declining years of the honored old veteran, let him address Dr. S. G. Perry, 46 West 37th street, New York City.—*Dental Pract. and Advertiser.*

---

ARTICLE V.

